# Caring During COVID-19: Inequalities in Resource- and Information-Sharing by Race, Ethnicity, and Nativity

**DOI:** 10.1093/geroni/igab046.646

**Published:** 2021-12-17

**Authors:** Sung Park

**Affiliations:** Princeton University

## Abstract

Americans experienced significant challenges as a result of the pandemic, further magnifying the weak U.S. social safety net. With few institutional supports available, individuals turned to each other for assistance. Relying on multiple nationally representative surveys, this study examines resource-sharing by race, ethnicity, and nativity over a one-year period during COVID-19. Furthermore, this study examines knowledge-related behaviors, such as information-seeking and information-sharing, which were also important tools utilized during the pandemic. Differences in both resource- and information-sharing contributed to disparities in the perceptions of risk, the reported levels of need, and concomitant behaviors linked to well-being. This research emphasizes the importance of personal relationships during times of crisis, and the role of social connections in shaping health and economic inequalities between minority- and non-minority populations.

